# Crack Cocaine Use-Related Spinal Cord Infarct

**DOI:** 10.7759/cureus.45207

**Published:** 2023-09-14

**Authors:** Srikanth Adidam Venkata, Narek Hakobyan, Huan Yang, Artem Sunik, Amit Khaneja

**Affiliations:** 1 Neurology, Downstate Neurology at One Brooklyn Health, Brooklyn, USA; 2 Neurology, State University of New York Downstate Health Sciences University, Brooklyn, USA; 3 Internal Medicine, Brookdale University Hospital Medical Center, Brooklyn, USA; 4 Neurology, Brookdale University Hospital Medical Center, Brooklyn, USA

**Keywords:** sudden onset paraplegia, spine imaging, stroke, spinal cord ischemia, crack cocaine

## Abstract

In this study, we describe an unusual occurrence of spinal cord infarct associated with acute usage of crack cocaine. A 64-year-old male patient was brought to the hospital after being found down, displaying weakness in his lower extremities and positive for cocaine use on a urine toxicology test. The patient was administered intravenous fluids and evaluated for syncope and rhabdomyolysis. Upon initial medical assessment, the patient exhibited sensation loss up to the level of the mid-thigh, paraplegia, urinary retention, and decreased rectal sphincter tone. Neurological examination and neurological imaging were suggestive of acute spinal cord infarct.

## Introduction

Acute spinal cord ischemia (ASCI) is a rare but devastating condition that involves the sudden interruption of blood supply to the spinal cord, leading to neurological deficits and significant morbidity. Although various etiologies have been implicated in ASCI, the association between crack cocaine use and spinal cord ischemia has emerged as a concerning phenomenon in recent years. Crack cocaine, a potent and highly addictive stimulant derived from powdered cocaine, has become a widespread illicit drug of abuse. Its use has been associated with various adverse effects, including cardiovascular complications, neurologic deficits, and ischemic events in multiple organ systems. The link between crack cocaine use and ASCI has gained recognition due to the drug's profound vasoconstrictive properties, its ability to disrupt normal hemodynamic regulation, and the formation of thromboembolic events [1]. 

The clinical presentation of acute spinal cord ischemia associated with crack cocaine use can vary widely, often mimicking other spinal cord pathologies or drug-related effects. Early recognition and prompt intervention are essential to minimize neurological damage and optimize outcomes. However, the diagnosis of ASCI remains challenging due to the lack of specific clinical signs and the absence of sensitive diagnostic tools. Improved understanding of the pathophysiology, clinical manifestations, and treatment strategies will facilitate early identification, appropriate intervention, and ultimately improve outcomes for patients affected by acute spinal cord ischemia in the setting of crack cocaine use.

## Case presentation

A 64-year-old male with past medical history of hypertension, a history of drug abuse, and a past surgical intervention involving L4-L5 lumbar laminectomy and fixation. He was found fallen next to paraphernalia related to crack cocaine use. Subsequently, he was transported to the hospital by emergency medical service (EMS) for a comprehensive examination.

Upon admission, the patient's vital signs included a blood pressure of 126/88 mmHg, respiratory rate of 16 breaths per minute, body temperature of 36.8 degrees Celsius, a pulse rate of 88 beats per minute, and an oxygen saturation level of 98% while breathing ambient air. The patient reported consuming crack cocaine, subsequently losing consciousness, and regaining awareness on the floor, unable to mobilize his legs. He also reported an inability to void urine or defecate since the incident.

A neurological evaluation revealed the patient to be paraplegic with hypotonia in both lower extremities, alongside a diminished response to reflex testing at both patellas and Achilles tendons. Sensation to soft touch and pinprick tests was absent below the umbilical level. Although proprioception was retained at the hips, the patient demonstrated inconsistent sensation to vibration throughout both lower extremities. Furthermore, the patient exhibited decreased rectal tone, and his abdominal examination showed signs of distension and succussion splash, indicative of urinary retention. As a result, a Foley catheter was inserted.

**Figure 1:**
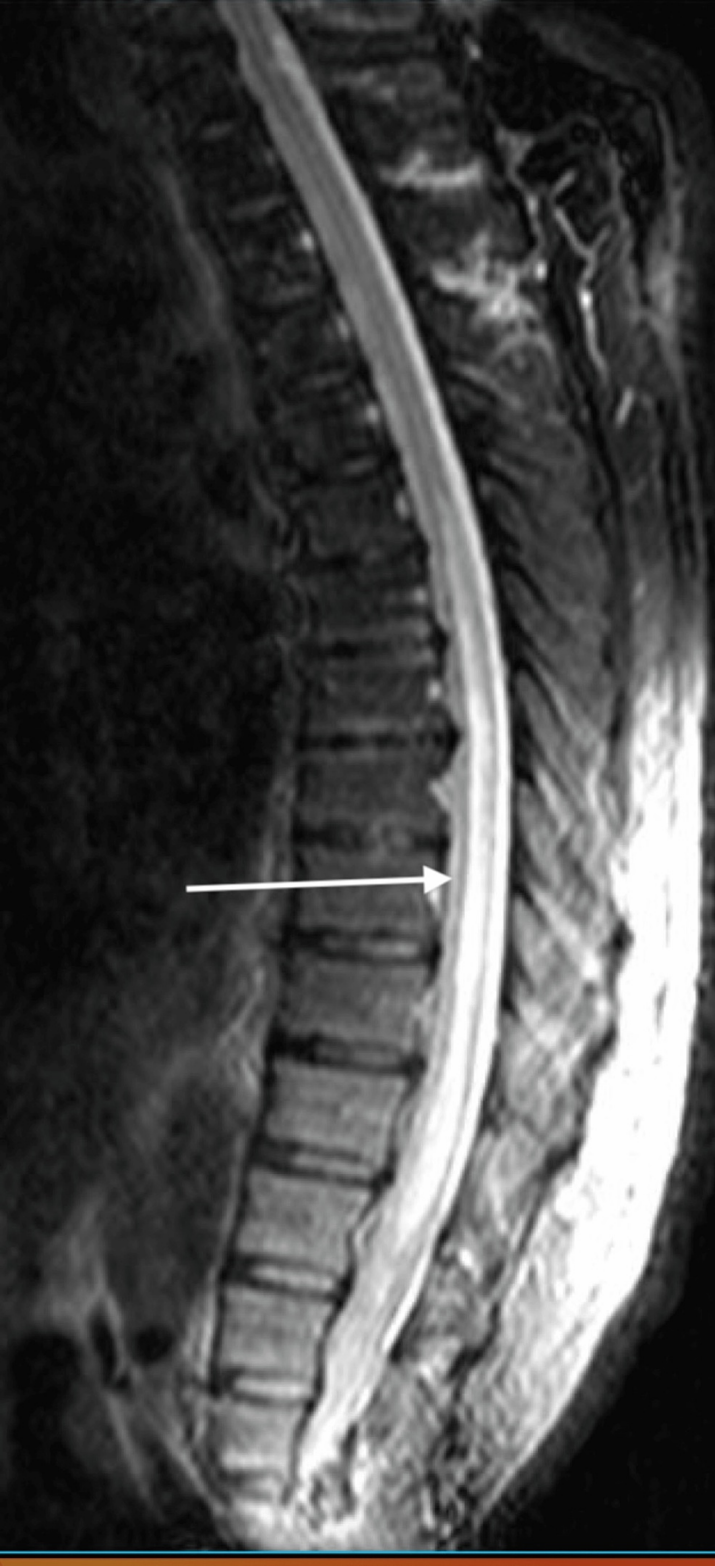
Magnetic resonance imaging of the thoracic spine with fat suppression showing hyperintensity from T9 to conus

His laboratory results revealed a leukocyte count of 13,800 cells per cubic millimeter (normal range: 4,300 and 10,800 cells per cubic millimeter), indicative of leukocytosis, and a significantly elevated creatine kinase level of 1238 micrograms per liter (normal range: 10 to 120 micrograms per liter). An initial magnetic resonance imaging (MRI) of the brain scan was unremarkable; however, an MRI of the thoracic spine (Figure 1) revealed an abnormal hyperintensity from T9 to the conus medullaris.

**Figure 2:**
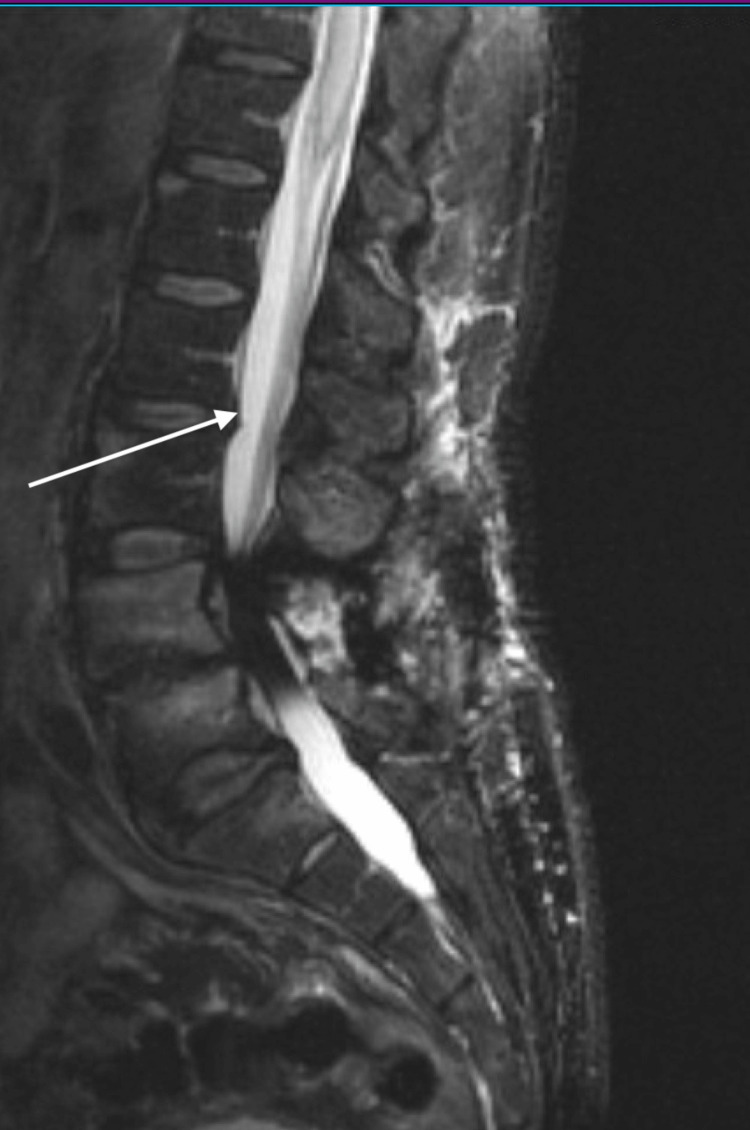
Magnetic resonance imaging of the lumbar spine showing abnormal hyperintensity from the T9 segment to the conus

In addition, the MRI of the lumbar spine (Figure 2) also showed an abnormal hyperintensity from T9 to the conus medullaris. 

**Figure 3:**
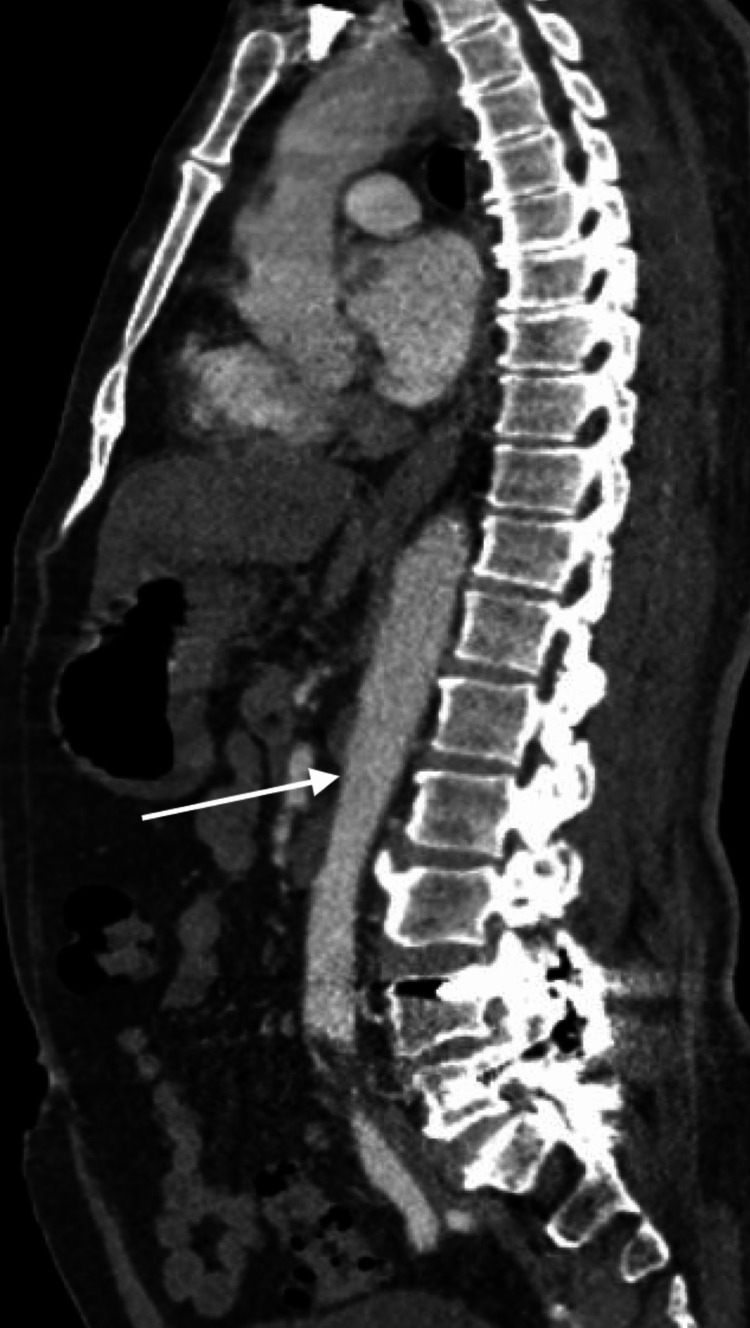
Computed tomography angiography of the chest, abdomen, and pelvis demonstrating normal thoracic and abdominal aorta

The findings from both magnetic resonance imaging studies strongly indicated the presence of an acute spinal cord infarction or transverse myelitis. Computed tomography angiography (CTA) of the chest, abdomen, and pelvis was conducted and reviewed by radiology, vascular and cardio-thoracic surgery, interventional radiology, interventional neurology, and neurosurgery who ruled out thoracic aortic dissection and stroke of the artery of Adamkiewicz (Figure 3).

The patient rejected lumbar drain placement to increase spinal perfusion pressures after psychiatry cleared him for capacity. Given the diagnostic uncertainty between transverse myelitis and acute ischemic events affecting the spinal cord, a therapeutic approach combining aspirin at a daily oral dose of 81 milligrams and intravenous corticosteroids for a five-day period was initiated. 

**Figure 4:**
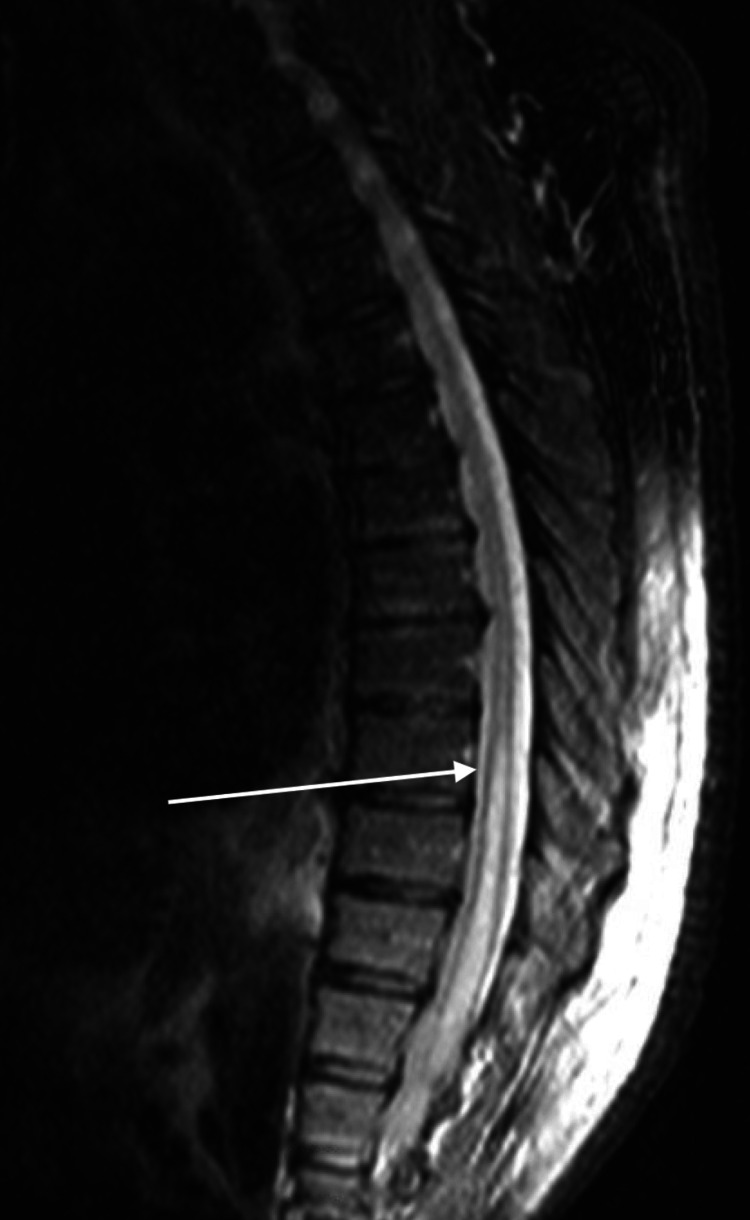
Repeat magnetic resonance imaging of the thoracic spine with fat suppression showing hyperintensity from T5 to conus

Repeat MRI of the thoracic spine with fat suppression showed caudal extension of the lesion to T5 (Figure 4). 

**Figure 5:**
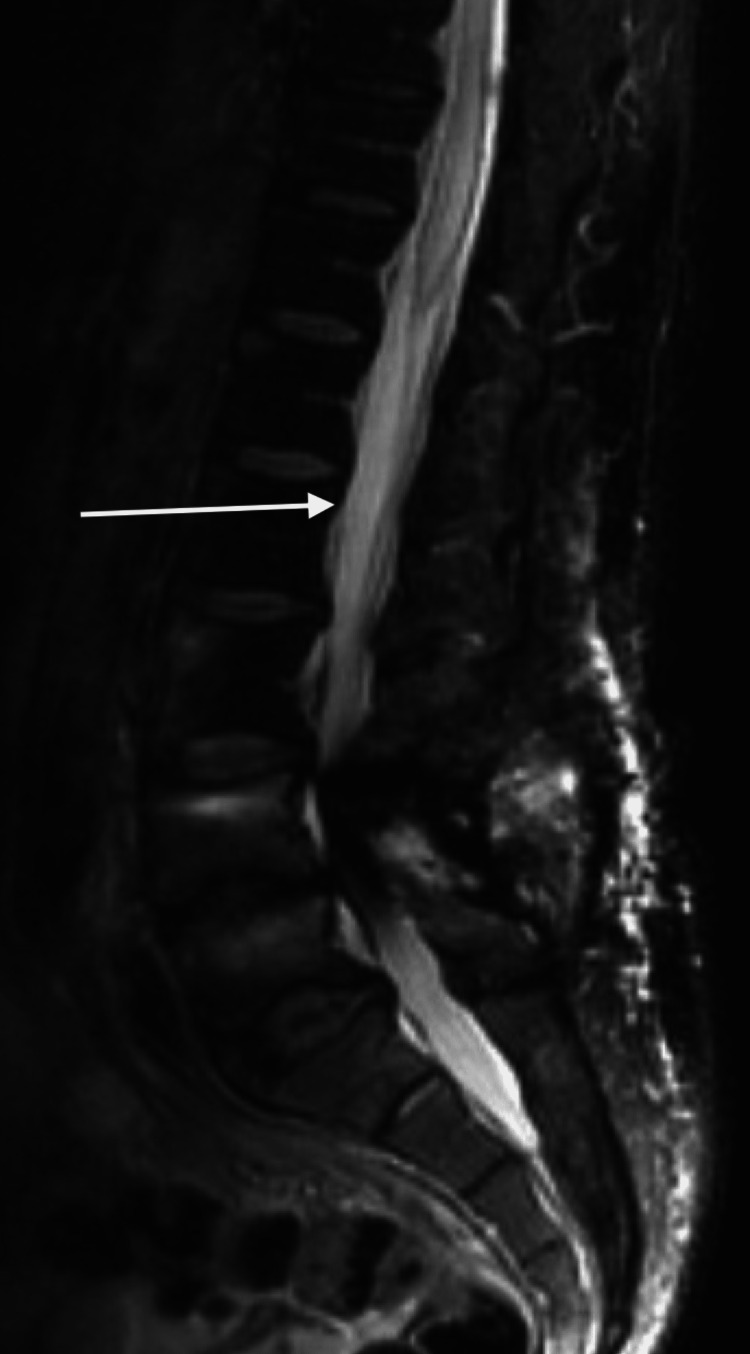
Magnetic resonance imaging of the lumbar spine also showing interval change of extension of the lesion caudally to T5

Repeat MRI of the lumbar spine with fat suppression also showed extension of the lesion caudally to T5 (Figure 5). 

A lumbar puncture was performed, and cerebrospinal fluid analysis (Table 1) did not confirm a pattern of infective or autoimmune pathology. 

**Table 1:** Cerebrospinal fluid analysis results

Laboratory test	Results	Reference range
White blood cell count	49 cells	0-5 cells per microliter
Red blood cell count	585.0 cells	Negative
Protein	148 mg/dL	12-60 mg/dL
Glucose	72 mg/dL	40-70 mg/dL
Neutrophil %	75%	40-60%
Oligoclonal Bands	Negative	Negative

Upon concluding the diagnostic investigations, the patient was discharged to a specialized acute rehabilitation facility. A prompt follow-up appointment with the neurology outpatient department was also scheduled.

## Discussion

This case report highlights a case of acute spinal cord ischemia (ASCI), an infrequent but critical condition that is often challenging to diagnose [2]. Our patient, with a significant medical history and evident drug abuse, presented with rapid-onset paraplegia following an episode of substance abuse. 

Cocaine, especially in its smokable form (crack), is known to induce strong systemic vasoconstriction, secondary to its inhibitory effects on the reuptake of norepinephrine at sympathetic nerve endings. This is hypothesized to affect the blood vessels supplying the spinal cord [3]. Cocaine use has been associated with systemic inflammation and oxidative stress. These processes could exacerbate the vulnerability of the spinal cord to ischemia by damaging the endothelial lining of the blood vessels, increasing the tendency for thrombus formation and reducing the oxygen-carrying capacity of the blood [4]. 

Acute spinal cord ischemia typically manifests as sudden-onset motor and sensory deficits, as evidenced in our patient's presentation [5]. A key differential diagnosis to consider in such cases is transverse myelitis (TM) [6]. 

To establish the correct diagnosis, a comprehensive diagnostic workup is necessary. Imaging studies, including magnetic resonance imaging (MRI) of the spine, play a crucial role in differentiating between ASCI and TM. In ASCI, MRI typically reveals hyperintensity on T2-weighted images, which may span multiple spinal segments [7], indicating ischemic injury. In TM, there is often a longitudinally extensive lesion spanning multiple spinal segments with variable enhancement patterns. Cerebrospinal fluid (CSF) analysis may demonstrate pleocytosis and increased protein levels in both conditions [8], but the lack of oligoclonal bands was more suggestive of ASCI.

Management in this scenario involved a therapy regimen of aspirin and IV corticosteroids, which aimed at reducing further ischemic risk and managing potential inflammatory responses [9]. Following the completion of the immediate therapeutic interventions, the patient was discharged to a rehabilitation facility, highlighting the crucial role of early rehabilitative intervention in improving functional outcomes [10].

## Conclusions

This case reinforces the significance of considering acute spinal cord ischemia in the differential diagnosis of acute paraplegia, especially in patients with relevant risk factors such as substance abuse. It also underscores the value of a multidisciplinary approach involving early therapeutic intervention and rehabilitation in managing such cases.
